# High Mannose Binding Lectin (PFL) from *Pseudomonas fluorescens* Down-Regulates Cancer-Associated Integrins and Immune Checkpoint Ligand B7-H4

**DOI:** 10.3390/cancers11050604

**Published:** 2019-04-30

**Authors:** Yuichiro Sato, Kiminori Matsubara, Takanori Kubo, Hirobumi Sunayama, Yuta Hatori, Kinjiro Morimoto, Toshio Seyama

**Affiliations:** 1 Department of Medical Pharmacy, Faculty of Pharmacy, Yasuda Women’s University, Hiroshima 731-0153, Japan; mori-k@yasuda-u.ac.jp; 2 Department of Human Life Science Education, Graduate School of Education, Hiroshima University, Higashi-Hiroshima 739-8524, Japan; kmatsuba@hiroshima-u.ac.jp; 3 Department of Life Sciences, Faculty of Pharmacy, Yasuda Women’s University, Hiroshima 731-0153, Japan; kubo-t@yasuda-u.ac.jp (T.K.); hatori@yasuda-u.ac.jp (Y.H.); seyama@yasuda-u.ac.jp (T.S.); 4 Department of Pharmaceutical Chemistry, Faculty of Pharmacy, Yasuda Women’s University, Hiroshima 731-0153, Japan; sunayama@yasuda-u.ac.jp

**Keywords:** lectin, integrin, autophagy, angiogenesis, immune checkpoint

## Abstract

*Pseudomonas fluorescens* lectin (PFL), which belongs to the high mannose (HM)-binding OAAH (*Oscillatoria agardhii* agglutinin homologue) lectin family, induces cancer cell death. However, the detailed mechanisms underlying this process have not yet been elucidated. We found that PFL decreased various integrins as well as EGFR in cancer cells by promoting internalization and autophagic degradation of these molecules, subsequently inducing caspase-8 dependent cell apoptosis. As revealed by an ex vivo angiogenesis assay using the rat aortic model, PFL inhibited neovascularization in a dose-dependent manner, which was potentially mediated by down-regulation of endothelium integrins. Interestingly, PFL also down-regulated B7-H4 in cancer cells, which has been implicated as a negative regulator of T cell-mediated immunity. We found that B7-H4 co-localized with β3 integrin in MKN28 gastric cancer cells. siRNA silencing of B7-H4 in MKN28 cells decreased expression of β3 integrin, suggesting physical and functional association between these molecules. Direct interaction of PFL with integrin αvβ3 or B7-H4 was examined by surface plasmon resonance analysis, which detected high affinity glycan-dependent binding to PFL. These investigations suggest that PFL interaction with cell surface integrins is a key process for the anti-cancer activities of PFL.

## 1. Introduction

Integrins are heterodimeric glycoproteins that mediate cell adhesion to the extracellular matrix and immunoglobulin superfamily molecules. Expressions of a wide variety of integrins, such as αvβ3, αvβ5, α5β1, and α6β4, on cancer cells have been reported to correlate with cancer progression, metastasis, and poor survival [1,2]. Specific endothelial cell integrins, such as αvβ3, α5β1, and αvβ5, also play important roles in angiogenesis by binding provisional matrix proteins in the tumor environment [3]. Thus, various integrin antagonists including small peptidic or peptidemimetic molecules, such as cilengitide or antibody-based compounds, are being developed for potential clinical application [4]. A growing body of evidence suggests that integrin crosstalk with growth factor receptors and cytokine receptors results in cooperative signaling that affects tumor cell proliferation, motility, survival, and metastasis [1]. Depending on the cellular and environmental context, integrins form a molecular complex with growth factor receptors. For example, integrin β4 is physically associated with ErbB2 in breast cancer cells and thereby contributes to the growth and invasion of tumors [5]. It has also been reported that integrin β5 forms a complex with epidermal growth factor receptor (EGFR) and contributes to pancreatic cancer metastasis [6].

Notably, integrin glycans play important functions not only as crosslinkers to form complexes with growth factor receptors, but also as regulators controlling various aspects of integrin-mediated cell behavior, such as receptor clustering, and cell growth and migration [7,8]. Importantly, integrin N-glycans have been suggested to regulate conformational equilibria and ligand affinity [9]. Other endogenous molecules, such as galectin-3, are also likely to be involved in the formation of the α6β4-EGFR complex in cancer cells [7].

Contrastingly, a wide variety of exogenous lectins have potent anti-cancer activity [10]. The recently discovered high mannose (HM) binding lectin family from lower organisms also exhibits an anti-tumor effect. This family, referred to as the OAAH (*Oscillatoria agardhii* agglutinin homologue) lectin family, includes the cyanobacterial lectin OAA from *O. agardhii* [11] and other homologous proteins, such as bacterial lectin *Pseudomonas fluorescens* lectin (PFL) from *P. fluorescens* pf0-1 [12]. These lectins exclusively bind to HM-type N-glycans, which have a Manα(1-3)Manα(1-6)Man core as an essential recognition unit [11,12,13]. In a previous study, surface glycoproteins on MKN28 gastric cancer cells that directly interact with PFL were isolated and identified as integrin α2 and EGFR [12,14]. Following the glycan-dependent binding of PFL, these molecules were degraded by autophagy, sensitizing cancer cells to anti-cancer drugs [14]. However, the detailed mechanisms by which PFL induces cancer cell death remain incompletely understood.

In the present study, we found that in cancer cells, PFL led to a dramatic decrease in the levels of various integrins. PFL also had anti-angiogenic activity, which may have been modulated by integrin down-regulation in endothelial cells. Moreover, our results suggest an association between particular integrins, such as β3, and the immune check point ligand B7-H4. Up-regulation of these molecules has been observed in the tumor microvascular endothelium. Interestingly, PFL significantly decreased protein abundance of B7-H4 in a glycan-dependent manner. Therefore, our studies raise the possibility that carbohydrate-binding molecules regulate immune checkpoints by interacting with heavily glycosylated ligands such as B7-H4.

## 2. Results

**Figure 1:**
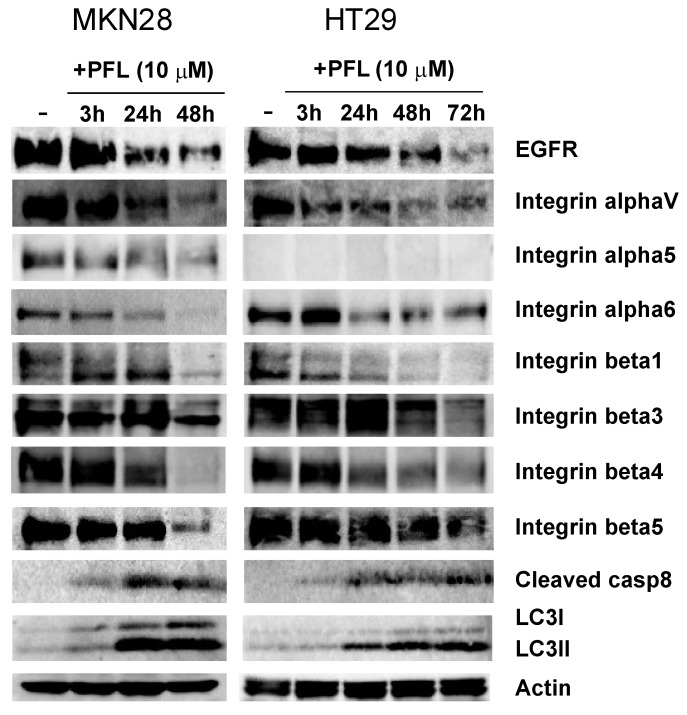
Effect of *Pseudomonas fluorescens* lectin (PFL) treatment on the abundance of multiple integrin subunits in MKN28 and HT29 cells. MKN28 and HT29 cells were incubated with 10 μM PFL in RPMI-1640 containing 10% fetal bovine serum (FBS) for 3, 24, and 48 h, or 3, 24, 48, and 72 h. The cells were lysed and subjected to immunoblot analysis. Protein levels were detected by immunoblotting with a monoclonal antibody against each protein. The figure shows results of one experiment that was replicated at least twice with similar results.

Our previous study demonstrated that the HM binding lectin PFL directly bound to cell surface α2 integrins and EGFR, and promoted internalization of these molecules [12,14]. However, the effect of PFL on other cancer-related integrins remains largely unknown. To investigate the effect of PFL on integrins in MKN28 gastric cancer cells and HT29 colon cancer cells, we examined expression levels of various integrin subunits after PFL treatment by western blotting. As shown in Figure 1, integrins such as αV, α6, β1, β3, β4, and β5 were significantly decreased by 10 μM PFL in a time-dependent manner, regardless of the cell line used. In addition, PFL decreased the level of α5 integrin in MKN28 cells, but α5 expression was not observed in HT29 cells. As previously reported, EGFR was also significantly reduced by PFL treatment. By contrast, the autophagic marker LC3II appeared at 24 h post-PFL treatment in both cell lines, indicating that autophagic degradation of cellular proteins was activated. In addition, enhanced levels of cleaved caspase-8 in cells treated with PFL were observed.

**Figure 2:**
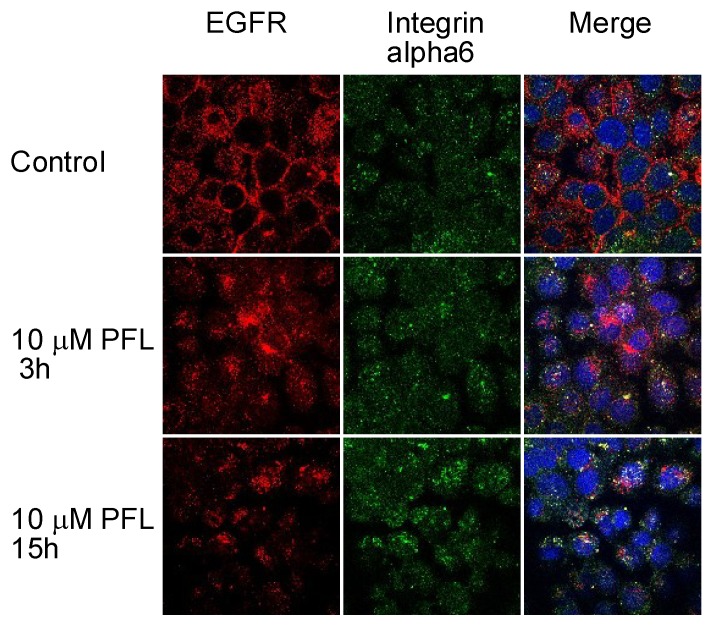
Cellular distribution of integrin α6 and EGFR with PFL treatment. MKN28 cells were incubated with 10 μM PFL for the indicated time. Integrin α6 and EGFR were visualized by confocal fluorescence microscopy. Representative images depict the cellular localization of integrin α6 (green) and EGFR (red). Co-localization of both proteins is represented as a yellow signal (merged panel). The figure shows results of one experiment that was replicated at least twice with similar results.

**Figure 3:**
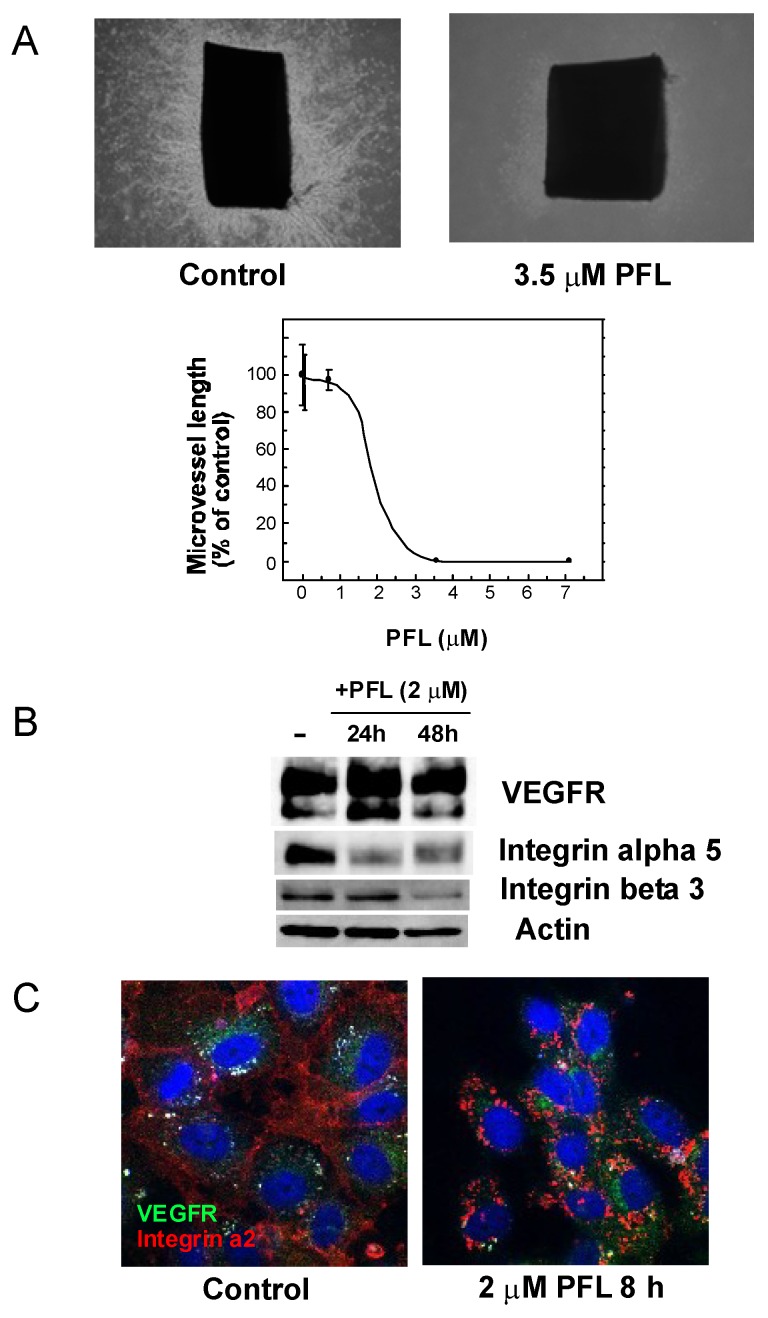
PFL inhibition of angiogenesis. (**A**) Effect of PFL on ex vivo angiogenesis of rat aortic rings. Representative data of PFL inhibition of angiogenesis are shown. The figure shows results of one experiment that was replicated at least twice with similar results. Rat aortic rings were embedded in collagen gel and maintained at 37 °C for 7 days in the presence or absence of various concentrations of PFL. Microvessel length was measured using Adobe Photoshop software CS3. Each value represents the mean and SD (*n* = 6). (**B**) Change of VEGFR integrin levels in human umbilical vein endothelial cells (HUVECs) treated with PFL. HUVECs were incubated with 2 μM PFL in HUVEC medium for 24 and 48 h on a vitronectin-coated plate with VEGF stimulation. Protein levels were detected by immunoblotting with a monoclonal antibody against each protein. (**C**) Cellular distribution of VEGFR and integrin α2 after PFL treatment. Altered cellular distribution of VEGFR (green) and integrin α2 (red) with PFL treatment was observed by immunofluorescence microscopy. HUVECs were incubated with 2 μM PFL in HUVEC medium for 8 h on vitronectin-coated cover glass with VEGF stimulation. Nuclei were stained with DAPI.

Subsequently, the effect of PFL on cellular distribution of integrin α6 and EGFR was examined. Immunofluorescence microscopy revealed that a large proportion of integrin α6 molecules co-localized with EGFR on MKN28 cells (Figure 2). It seems that EGFR coupled with integrin α6 was more resistant to PFL treatment, as most of the yellow signal was retained 15 h post-PFL treatment. Numerous studies have implicated certain integrins including αvβ3 and α5β1 regulate tumor angiogenesis and lympangiogenesis, thereby affecting tumor development and metastasis. As antagonists of these integrins are potential inhibitors of neovascularization, we next determined the antiangiogenic activity of PFL by an ex vivo angiogenesis model using rat aortic rings. As shown in Figure 3A, robust microvessel outgrowth from aortic rings was observed in the control groups. By contrast, PFL inhibited the outgrowth of microvessels in a dose-dependent manner, and 3.5 μM PFL completely inhibited angiogenesis. To gain insight into the mechanisms of PFL inhibition of angiogenesis, integrin levels were measured in human umbilical vein endothelial cells (HUVEC) treated with PFL. Integrins such as α5 and β3 were significantly decreased by PFL treatment in HUVECs, while vascular endothelial growth factor receptor (VEGFR) was not affected (Figure 3B). It has been reported that VEGFR and β3 integrin form a complex in the presence of vitronectin, an extracellular matrix protein that serves as a ligand for αvβ3 integrin [15]. We tested VEGFR levels using cells cultured on a vitronectin-coated plate stimulated with vascular endothelial growth factor (VEGF), but no significant change was observed with PFL treatment. Data obtained by immunofluorescence staining also indicated that the level of VEGFR was unaffected by PFL.

Irrespective of the presence of vitronectin, PFL affected the localization of integrins, but not that of VEGFR (Figure 3C). These results suggest that PFL inhibition of angiogenesis was possibly due to integrin down-regulation in endothelial cells.

**Figure 4:**
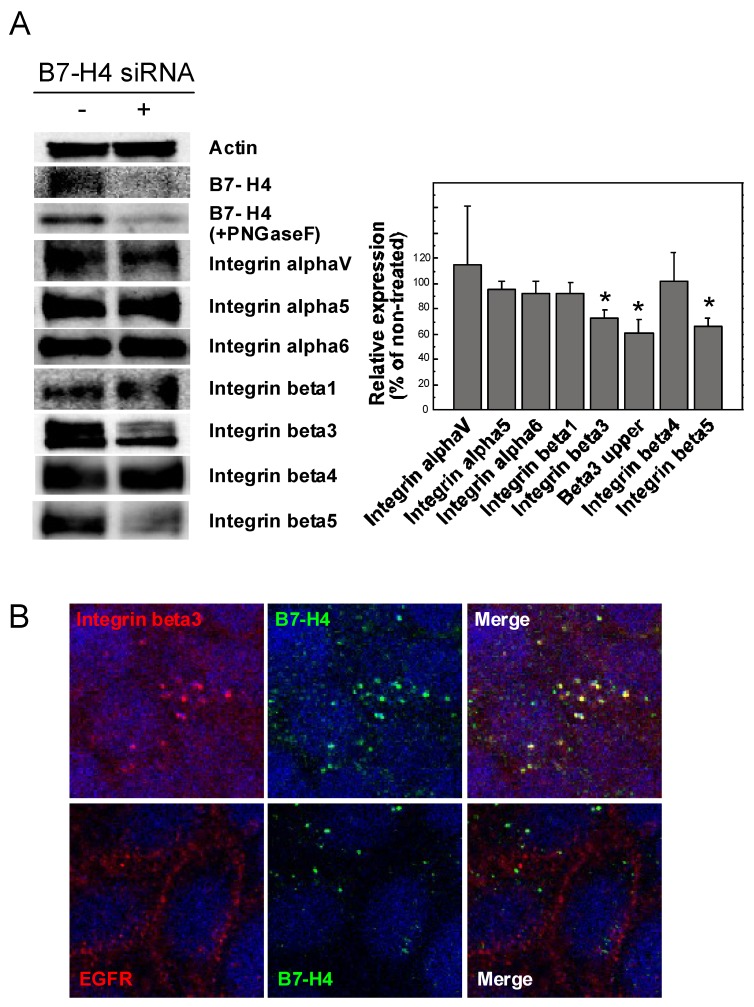
Survey of association between B7-H4 and integrins. (**A**) Effect of B7-H4 siRNA on levels of various integrins in MKN28 cells. After 48 h of B7-H4 siRNA treatment, integrin levels were assessed by western blot analysis. The value of relative protein levels is indicated in the right panel. The data in the bar graph represent combined data from two experiments, one of which is shown in the immunoblot representations. Asterisks indicate values significantly different (*p* < 0.01) compared with the non-treated control. (**B**) Co-localization of B7-H4 and integrin β3. Cellular distribution of B7-H4 (green) and integrin β3 (red) was observed by immunofluorescence microscopy (upper panels). Differential cellular distribution of B7-H4 (green) and EGFR (red) was also observed (lower panels). Co-localization of both proteins is represented as a yellow signal (merged panel). Nuclei within the cells were stained with DAPI.

In certain tumors, expression of specific glycoproteins, such as B7-H4, within the tumor microvascular endothelium has been observed [16]. B7-H4, known as a negative regulator of T cell-mediated immunity, has been implicated in tumor neovascularization, but its specific function remains unknown. To investigate the possibility of functional associations between B7-H4 and angiogenesis-related integrins, we examined the effect of B7-H4 siRNA knockdown on integrin expression in MKN28 cells. As shown in Figure 4A, B7-H4 knockdown decreased abundance of some integrins, such as β3 and β5. Intriguingly, the higher molecular weight band of β3 integrin was much more susceptible to B7-H4 silencing compared with the lower band (Figure 4A), suggesting that B7-H4 may affect β3 integrin glycosylation. Cellular localization of B7-H4 in MKN28 cells was subsequently examined by immunofluorescence microscopy, revealing that β3 integrin co-localized with B7-H4, but not with EGFR (Figure 4B). These results suggest that B7-H4 and β3 integrin are mutually related by a physical association, and may contribute to tumorigenesis.

**Figure 5:**
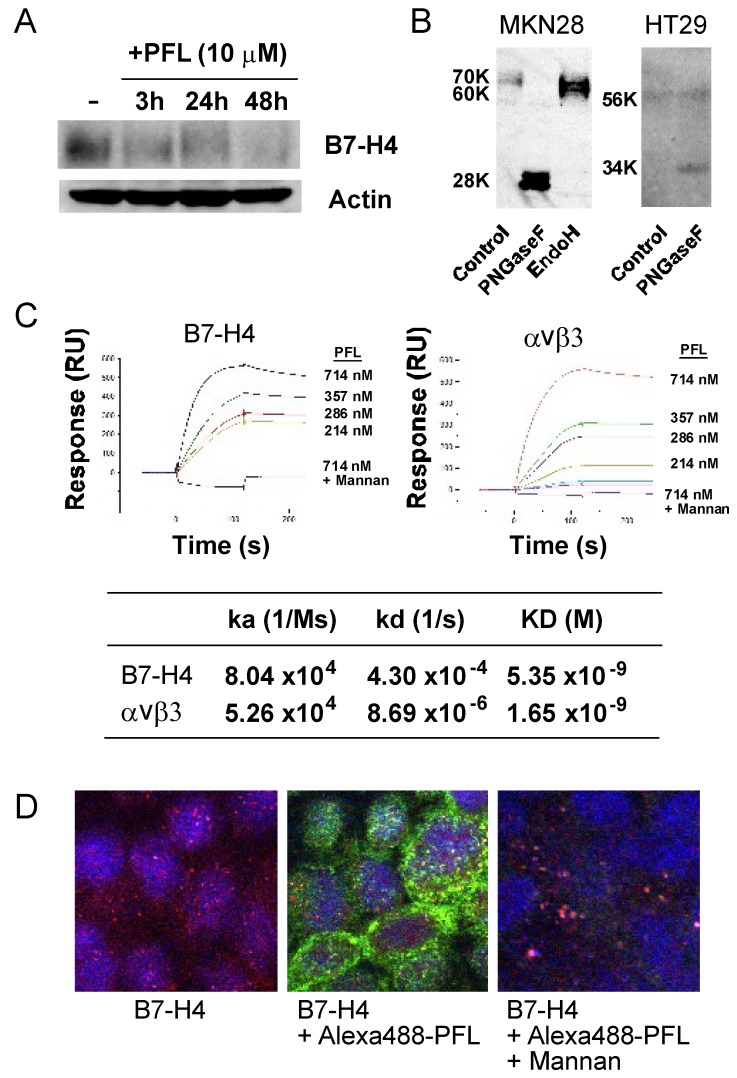
PFL decreases B7-H4 protein levels. (**A**) Decreased abundance of B7-H4 in MKN28 cells treated with PFL. MKN28 cells were incubated with 10 μM PFL in RPMI-1640 containing 10% FBS for 3, 24, and 48 h. The cells were lysed and subjected to western blot analysis. The figure shows results of one experiment that was replicated at least twice with similar results. (**B**) Removal of B7-H4 glycan by endoglycosidases. MKN28 and HT29 cell lysates were treated with PNGase F and Endo H, and subjected to western blot analysis. (**C**) Direct interaction between PFL and B7-H4 or αvβ3 was analyzed by surface plasmon resonance (SPR). Following immobilization of the ligand onto the CM5 sensor chip, the indicated concentrations of lectin solution were injected. Sensorgrams for binding were recorded in real time, and the response is expressed in resonance units (RU). Binding kinetics of the interaction between PFL and the ligand are shown. ka: association rate constant, kd: dissociation rate constant, KD: affinity constant. (**D**) Distribution of Alexa-488 PFL (green) and B7-H4 (red). Cellular localizations of Alexa-488 PFL and B7-H4 were examined with confocal fluorescence microscopy. Co-localization is shown as a yellow signal (Merge). Nuclei within the cells were stained with DAPI. The effect of yeast mannan (YM) (50 μg/mL) on PFL-B7-H4 interaction is also shown (right panel).

Next, we examined the effect of PFL on B7-H4 expression in MKN28 cells. Interestingly, PFL treatment significantly decreased B7-H4 protein (Figure 5A). Microarray analysis revealed that B7-H4 mRNA increased in the presence of PFL in a time-dependent manner (Table S1). The discrepancy between B7-H4 protein and mRNA levels may be a compensatory effect for decreased protein abundance. To determine if PFL down-regulation of B7-H4 is dependent on glycans, we first investigated the glycosylation status of B7-H4 in MKN28 cells. In MKN28 cells, intact 70 kDa B7-H4 was reduced to 28 kDa after treatment with PNGaseF, which cleaves all N-glycans, while treatment with Endo H, which cleaves HM glycans, shifted the B7-H4 band to 60 kDa (Figure 5B). The extent of glycosylation and protein expression of B7-H4 was different in various cancer cell lines. For instance, the amount of B7-H4 in HT29 cells was much lower than that of MKN28 cells (Figure 5B).

To explore glycan-mediated binding of PFL to B7-H4, the direct interaction between PFL and recombinant B7-H4 was evaluated by surface plasmon resonance (SPR) analysis. As shown in Figure 5C, PFL bound to immobilized B7-H4 in a dose-dependent manner. High affinity interaction (KD = 10^−9^) was detected between PFL and B7-H4. The binding affinity of B7-H4 was comparable to that of αvβ3 integrin. This interaction was effectively inhibited by yeast mannan bearing HM glycans, indicating that PFL bound to B7-H4 through HM glycans. Because the glycan structure of recombinant B7-H4 may differ from that of native B7-H4, direct interaction between PFL and B7-H4 was confirmed in MKN28 cells by immunofluorescence microscopy. PFL bound to cell surfaces and co-localized with B7-H4 (Figure 5D). As expected, these interactions were abrogated by mannan.

## 3. Discussion

We previously reported that PFL induced cell death accompanying EGFR/integrin internalization and subsequent autophagy in MKN28 gastric cancer cells [14]. In the present study, we provide further evidence that PFL down-regulates various cancer-associated integrins not only in MKN28 cells, but also in HT29 colon cancer cells. Enhanced levels of cleaved caspase-8 in PFL treated cells suggest that PFL-mediated cell death may occur via integrin-mediated death, a process known as unligated integrin-induced apoptosis. Moreover, PFL significantly inhibited angiogenesis, which we suggest is mediated by decreased endothelial cell integrins. Importantly, PFL decreased protein levels of B7-H4, also known as B7x, B7S1, or VTCN1, which has been implicated as a negative regulator of T cell-mediated immunity [17]. In some tumors, B7-H4 has been suggested to contribute to tumor neovascularization, as specific expression of B7-H4 in the tumor microvascular endothelium has been observed. For instance, B7-H4 but not B7-H1 (PD-L1) expression was observed within renal cell carcinoma tumor vasculature in most patient samples [16]. In the present study, we found that B7-H4 co-localized with integrin β3 in MKN28 cells. siRNA silencing of B7-H4 decreased integrin β3 protein levels, suggesting a potential physical and functional association of these molecules. However, the role of this interaction in tumor neovascularization remains to be elucidated. In addition, expression of angiogenesis-related integrin β5 was significantly decreased by B7-H4 knockdown, further suggesting a possible relationship between B7-H4 and integrins important in tumor neovascularization.

In MKN45 gastric cancer cells, a molecular complex comprised of integrin α6β4, EGFR, and galectin-3 has been reported [7]. Cancer cells are thought to promote migration through the phosphorylation of integrin β4, which is regulated by various RTKs, including EGFR [18]. We found that PFL decreased protein levels of integrin α6, β4, and EGFR in both MKN28 and HT29 cells, but it is unclear whether PFL binds to the glycans of each molecule independently, or to those of the entire molecular complex. Immunofluorescence microscopy revealed that integrin α6 and EGFR co-localized in MKN28 cells and were not dissociated after PFL treatment. It is therefore likely that PFL bound to the molecular complex comprising integrin α6 and EGFR, inducing degradation of the complex.

Increased levels of integrin α6β4 have been implicated in malignant progression and poor prognosis in various cancer types [7]. Previous studies demonstrated that integrin α6β4 was also associated with epithelial to mesenchymal transition (EMT), wherein cancer cells lose cell–cell adhesion and polarity, gaining invasive and metastatic properties [19]. Additionally, some integrins, including the transforming growth factor-β (TGF-β) activator αvβ6, are known to play a crucial role in EMT [20]. Further studies demonstrated that increased abundance of B7-H4 in intrahepatic cholangiocarcinoma up-regulated the mesenchymal marker vimentin and down-regulated the epithelial marker E-cadherin [21]. Although B7-H4 silencing did not alter the expression of integrin αv, α6, or β4 in the present study, cooperative regulation of EMT by B7-H4 and integrins should perhaps be explored in future studies. In this regard, TGF-β, a well-characterized EMT inducer, increases protein levels of B7-H4 in colorectal cells [22].

Some forms of neoplastic transformation cause profound changes in cellular glycosylation. Alterations in glycosylation in cancer cells could significantly impact a wide variety of cell surface receptors, directly influencing cellular signaling [23]. Recent findings emphasize that integrin N-glycans play a pivotal role in regulating integrin ligand affinity and conformational equilibria [9]. In the present study, we found that PFL drastically decreased various integrins in cancer cells, although additional mechanisms other than HM glycan binding may also mediate this decrease in integrin abundance. For example, lipid raft internalization upon cell detachment may be involved in this striking effect.

It should be noted that PFL significantly decreased protein abundance of the highly glycosylated immune check point ligand B7-H4. Recent work has also identified that non-glycosylated forms of the immune check point ligand B7-H1 (also known as PD-L1) undergo rapid protein degradation, which highlighted the importance of glycosylation in regulation of another immune check point ligand [24]. Aberrant glycosylation of B7-H3 in Ca9-22 oral cancer cells increases interaction with C-type lectin receptors such as DC-SIGN, suggesting the important role of immune check point ligand glycans in the modulation of immune recognition [25]. Given the very high specificity of PFL for HM glycans, our data indicate the presence of certain HM glycans on B7-H4. The physiological and functional role of bulk glycans in the regulation of B7-H4 will be elucidated in future studies.

Here, we provided the first evidence that B7-H4 was associated with specific integrins, such as integrin β3. Furthermore, we found that PFL decreases protein abundance not only of various cancer-related integrins but also of the immune checkpoint ligand B7-H4 in cancer cells. These findings provide new insights into lectin-mediated regulation of immune checkpoint molecules.

## 4. Materials and Methods 

### 4.1. Materials

Antibodies against integrins (αv, α5, α6, β1, β3, β4, and β5), the apoptosis-related protein cleaved caspase-8, and the autophagy marker LC3 were all purchased from Cell Signaling Technology (CST, Tokyo, Japan). Alexa-conjugated anti-mouse and anti-rabbit IgG were purchased from Life Technologies (Eugene, OR, USA). Recombinant integrin αvβ3 and B7-H4 were purchased from R&D Systems (Minneapolis, MN, USA). B7-H4 siRNA and non-targeting control siRNA were purchased from Dharmacon (Lafayette, CO, USA).

### 4.2. Cell Lines and Culture Conditions

The gastric cancer cell line MKN28 and the human colon adenocarcinoma cell line HT29 were obtained from Dr. Yanagihara at the National Cancer Center Research Institute, and were maintained in RPMI-1640 medium (Wako, Japan) supplemented with 10% fetal bovine serum (FBS, Gibco), 100 IU/mL penicillin G sodium, and 100 μg/mL streptomycin sulfate as described previously [14]. HUVECs were purchased from KAC (Amagasaki, Japan) and grown in HUVEC medium (DMEM/F12, 10% FBS, 10 ng/mL b-FGF) (DS Pharm Biomedical, Osaka, Japan).

### 4.3. Western Blotting

The abundance of cellular proteins was determined by western blotting. Confluent MKN28 and HT29 cells cultured in 6-well plates were incubated with 10 μM PFL in RPMI-1640 containing 10% FBS for 3, 24, and 48 h, or 3, 24, 48, and 72 h, respectively. Cells were then lysed with 800 μL RIPA buffer (50 mM Tris-HCl, pH 7.6, 150 mM NaCl, 1% Nonidet P40, 0.5% sodium deoxycholate, protease inhibitor cocktail, 0.1% SDS) for 20 min on ice. Portions of each sample were subjected to SDS-PAGE and western blotting. Proteins were detected using a specific rabbit antibody (1:1000) against each protein, as outlined in the materials section. Following incubation with horseradish peroxidase (HRP)-conjugated anti-rabbit IgG (1:10,000), proteins were visualized with ECL prime (GE Healthcare, Buckinghamshire, UK).

### 4.4. Ex Vivo Angiogenesis Assay

Male Wister rats (6 weeks old, Charles River Laboratories) were maintained according to guidelines established by the Hiroshima University Animal Research Committee (Approval No. C17-17). Briefly, the femoral artery of the rat was turned inside out and cut into segments of approximately 1.0–1.5 mm. The aortic segments were then placed in 6-well plates and covered with 0.5 mL of collagen gel solution consisting of eight volumes of porcine tendon collagen solution (3 mg/mL) (Cellmatrix Ia, Nitta Gelatin, Japan), one volume of 10× Eagle’s MEM (Gibco), and one volume of reconstitution buffer (80 mM NaOH and 200 mM HEPES). Following incubation at 37 °C for 20 min, the gel was overlaid with 2 mL of RPMI-1640 containing 1% ITS+ (BD Biosciences, Bedford, MA, USA), 50 IU/mL penicillin, and 50 μg/mL streptomycin and various concentrations of PFL or vehicle (PBS; 20 mM phosphate buffer, pH 7.0, 150 mM NaCl), and incubated at 37 °C for 7 days. Microvessel length was estimated by measuring the distance from the cut end to the midpoint of the vessel as described previously [26]. Each value represents the average of six culture samples.

### 4.5. Surface Plasmon Resonance Analysis

Direct interaction of PFL with integrin αvβ3 and B7-H4 was assessed using a BIAcore X100 system. The sensor chips (CM5) were activated with NHS (N-hydroxysuccinimide) and EDC (N-ethyl-N’-dimethylaminopropyl carbodiimide). Recombinant αvβ3 or B7-H4 was immobilized onto the sensor chip by a standard amine coupling method. The unreacted groups on the sensor surface were blocked with 1 M ethanolamine. Binding experiments were carried out using various concentrations of PFL at a flow rate of 30 μL/min with a running buffer (HBS-N) consisting of 10 mM HEPES and 150 mM NaCl (pH 7.4). Inhibition of PFL and ligand interaction was examined with 50 μg/mL yeast mannan. The conditions of the kinetics/affinity assay were as follows: contact time, 120 s; dissociation time, 600 s. The surface was regenerated by 10 mM glycine-HCl, pH 1.5. Kinetic parameters (ka, kd, and KD) were calculated by fitting the data to the Langmuir model for 1:1 binding using Biacore X100 evaluation software (BIAcore international AB, Uppsala, Sweden).

### 4.6. Cellular Distribution of Integrins and Other Molecules

The cellular distribution of integrins and EGFR with PFL treatment was assessed by immunofluorescence microscopy as described previously [14]. Confluent MKN28 cells growing on coverslips in a 6-well plate were incubated with 10 μM PFL in RPMI-1640 for various periods of time. The cells were fixed with 80% acetone and incubated with rabbit monoclonal anti-integrin α6 antibody (CST, Japan) and mouse monoclonal anti-EGFR antibody (Thermo Scientific, Cheshire, UK) at 37 °C for 1 h. After washing with PBS, the cells were incubated with Alexa 488-conjugated anti-rabbit IgG antibody and Alexa 568-conjugated anti-mouse IgG antibody (Life Technologies, Eugene, OR, USA) at 37 °C for 1 h. The cells were mounted using Vectashield with DAPI (Vector Laboratories, Burlingame, CA, USA) and observed using a confocal laser scanning microscope (IX70; Olympus, Tokyo, Japan).

The cellular distribution of integrin α2 and VEGFR in HUVEC with PFL treatment was examined in a similar manner. HUVECs growing on vitronectin-coated coverslips in a 6-well plate were incubated with 2 μM PFL in VEGF-containing medium for 8 h. The cells were fixed with 80% acetone and incubated with mouse anti-integrin α2 antibody and rabbit anti-VEGFR antibody (CST, Japan). According to the first antibody used, the corresponding secondary antibodies were employed.

The cellular localization of integrin β3 and B7-H4 in MKN28 cells was examined in a similar manner. Confluent MKN28 cells growing on coverslips in a 6-well plate were fixed with 80% acetone and incubated with mouse monoclonal anti-integrin β3 antibody (abcam, Tokyo, Japan) and rabbit monoclonal anti-B7-H4 antibody (CST, Japan). Visualization was performed as described above.

### 4.7. Effect of siRNA B7-H4 on Integrin Expression

MKN28 cells were adjusted to 5 × 10^4^ cells/mL in 1 mL RPMI-1640 per well of a 24-well plate and cultured. One hundred picomoles of B7-H4 siRNA was pre-incubated with 1.5 μL Lipofectamine RNAiMAX (LFRNAi; Thermo Fisher Scientific, Waltham, MA, USA) in 100 μL Opti-MEM (Thermo Fisher Scientific, Waltham, MA, USA) for 30 min. Subsequently, 900 μL Opti-MEM followed by 100 μL of the siRNA mixture was added to each well to obtain a final concentration of 100 nM siRNA. At 6 h post-transfection, the siRNA-containing culture medium was replaced with fresh RPMI-1640. Cells were then incubated with RPMI-1640 for 42 h, and cell extracts were prepared as described above. The protein level of B7-H4 was determined by western blotting using rabbit anti-B7-H4 antibody (CST, Japan) followed by HRP-conjugated anti-rabbit IgG (CST, Japan).

## 5. Conclusions

In this study, we demonstrated novel anti-cancer activities of exogenous lectin PFL with high mannose (HM) glycan binding specificity. PFL down-regulated various cancer-related integrins as well as immune check point ligand B7-H4, suggesting the importance of endogenous lectin for cancer cell behavior. Our results also indicate that B7-H4 might associate with particular integrins in certain tumor environments.

## Supplementary Materials

The following are available online at https://www.mdpi.com/2072-6694/11/5/604/s1, Table S1: Change of expression levels of immune checkpoint-related genes in MKN28 cells after PFL treatment.
