# Supplementary material for: High Mannose Binding Lectin (PFL) from Pseudomonas fluorescens Down-Regulates Cancer-Associated Integrins and Immune Checkpoint Ligand B7-H4
Source: Cancers (Basel). 2019 Apr 30;11(5):604. doi: 10.3390/cancers11050604 (PMC6562446; doi:10.3390/cancers11050604)
Supplement: Supplementary file 1 [file cancers-11-00604-s001.pdf]

Table S1 Change of expression levels of immune checkpoint-related genes in MKN28 cells after PFL treatment.

Gene	Description	Log2 Ratio		
		24 h	48 h	72 h
CD274	Homo sapiens CD274 molecule (CD274), mRNA [NM_014143]	1.00	0.65	0.26
VTCN1	Homo sapiens V-set domain containing T cell activation inhibitor 1 (VTCN1), mRNA [NM_024626]	0.62	1.86	2.15

Total RNA from PFL-treated and untreated MKN28 cells was extracted and subjected to Aligent Expression Array Analysis with SurePrint G3 Human GE 8x60K v2 (Takara Bio, Japan). The expression level of each gene at the indicated time was determined and compared with that of control RNA from PFL-untreated cells. The highlighting represents the change of the expression level by more than two fold.
